# Green Tea Polyphenol-Sensitive Calcium Signaling in Immune T Cell Function

**DOI:** 10.3389/fnut.2020.616934

**Published:** 2021-01-28

**Authors:** Yogesh Singh, Madhuri S. Salker, Florian Lang

**Affiliations:** ^1^Institute of Medical Genetics and Applied Genomics, Eberhard Karls University, Tübingen, Germany; ^2^Women's Hospital, Eberhard Karls University, Tübingen, Germany; ^3^Institute of Vegetative and Clinical Physiology, Eberhard Karls University, Tübingen, Germany

**Keywords:** EGCG, miR-15b, T cells, SOCE, Ca^2+^ influx

## Abstract

Polyphenol compounds found in green tea have a great therapeutic potential to influence multiple human diseases including malignancy and inflammation. In this mini review, we describe effects of green tea and the most important component EGCG in malignancy and inflammation. We focus on cellular mechanisms involved in the modification of T cell function by green tea polyphenol EGCG. The case is made that EGCG downregulates calcium channel activity by influencing miRNAs regulating expression of the channel at the post-transcriptional level.

## Biological Effects and Active Components of Green Tea

The global consumption of tea is estimated to be 273 billion L/year, and its putative impact on health has attracted considerable scientific interest (1–3). It is believed that green tea (*Camellia sinensis*) was first cultivated from China and has been manufactured and used for drinking purposes for several centuries (1). Green tea is the part of *Theaceae* plant family that encompasses several other plants and shrubs of medicinal and ornamental interest and is chiefly consumed in East Asia, the Indian subcontinent, and Southeast Asia (4). After water, green tea is probably the second most consumed beverage worldwide (4, 5). Green tea has health-promoting effects in a number of pathological disorders, such as cardiovascular disease, neurodegeneration, stroke, obesity, diabetes, and viral or bacterial infections (6–8). Furthermore, due to the anti-cancer properties of green tea, its components may be used for protection against cancer (9–15).

Tea is produced in various forms due to distinct manufacturing processes (4). Green tea is produced from fresh tea leaves; however, steaming or pan-frying process is used further for enzyme deactivation, which precludes the oxidation of polyphenols termed catechins present in the tea leaves (6, 12). Tea mainly contains catechins that roughly contribute 30–40% in brewed desiccated green tea including (–)-epigallocatechin-3-gallate (EGCG), (–)-epigallocatechin (EGC), (–)-epicatechin gallate (ECG), and (–)-epicatechin (EC) (5, 6, 12, 16–19). EGCG is the utmost catechin available in green tea and roughly embodies 50–80% of catechins in a 200–300 mg/brewed cup of green tea (20). EGCG is the best-studied green tea component and the principal polyphenol involved in health benefiting actions such as anti-inflammatory and anti-carcinogenic effects (12, 21).

## Influences of EGCG AND Related Substances

Green tea and its components were already demonstrated to counteract malignancy in several animal experiments (8, 9, 11, 22), but their biological activity in human subjects is still a matter of controversy (12, 23, 24). EGCG has been shown to affect angiogenesis and apoptosis, and acts as an antioxidant in different types of cancer and neurodegenerative diseases (6, 14, 20, 25). However, the significance of these findings was questioned, as most of the experiments performed in these studies had used a concentration range from 20 to 200 μM EGCG, which is higher than the serum concentration of EGCG encountered in humans (<10 μM) (12). The EGCG concentrations in human serum or plasma can be found in a range of 0.1 and 1 μM following drinking few cups of green tea and may approach 7 μM with supplements (12, 13, 26, 27).

Some reports have suggested that these dietary compounds may need some modification or changes in their structure to improve the safety and effectiveness so that they can achieve their maximum bioavailability and function (28–30). Therefore, EGCG has been modified by modulation of hydroxyl groups with peracetate groups called pEGCG (prodrug of EGCG, EGCG octa-acetate) to augment the bioavailability and stability of green tea polyphenol EGCG (12, 30, 31). The resulting polyphenolic compounds displayed enhanced anti-proliferative activity in breast cancer (12). A nanoparticle-based EGCG delivery system is already considered for oral dispensation in murine xenograft model (nude mice) with human prostate cancer (nanochemoprevention), resulting in 10 times dose advantage for pro-apoptotic and anti-angiogenic effects *in vitro* and *in vivo* (14).

The mechanism that causes the health-promoting properties of EGCG is the suppressive effect on growth of different cell types (1, 8, 11–13, 15, 22, 32–37). Conversely, the cell growth suppressed by EGCG is not only restricted to the tumor or cancerous cells, but it can also reduce the growth of cells that are not cancerous in nature such as bovine vascular smooth muscle cells (5). EGCG oxidizes easily and this can significantly affect its binding properties, thus impacting on cell adhesion ligand accessibility and matrix rigidity of cancer cells (38). In addition to several beneficial effects of green tea polyphenols, it can also have some potential side effects, which are summarized in recent reviews (27, 39). In brief, excessive consumption of green tea could lead to several side effects including dehydration (as green tea has diuretic property), deranged bile acid synthesis, gastroesophageal reflux disease and interference with iron metabolism (4, 39). Further research is warranted to investigate the beneficial and adverse effects of EGCG.

## Interaction Between Dietary Polyphenols and Gut Microbiome

**Figure 1:**
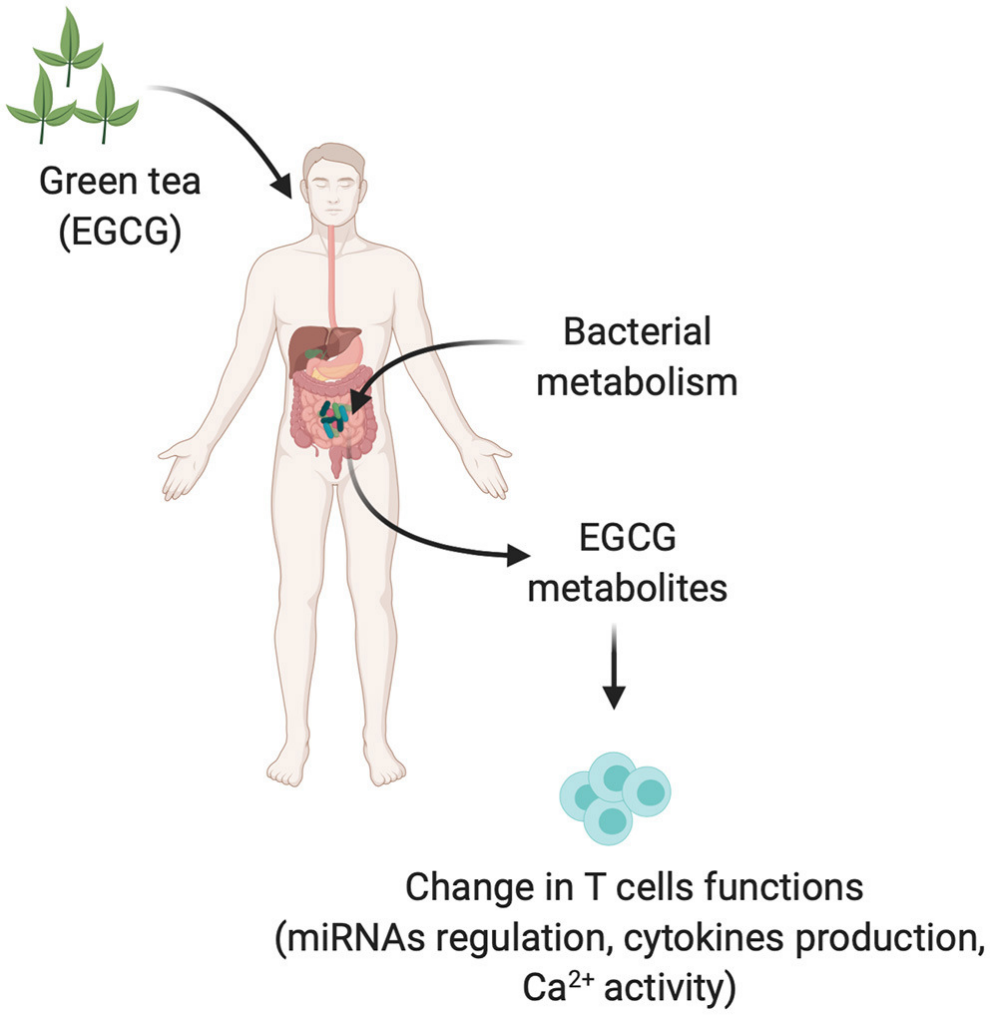
Gut microbiota in modulation of green tea into different metabolites and possible immune T cells dysregulation. EGCG and EGCG derived metabolites produced by gut microbiota could be modifying the effector functions of immune T cells by different mechanisms such as upregulating the miRNAs, cytokine production, or Ca^2+^ activity.

The interaction between polyphenols including their metabolites and gut microbiota is critical to understanding the biological mechanisms of polyphenols, since polyphenols are poorly absorbed and most of them are metabolized by the microbiome to form phenolic metabolites (40). Dietary polyphenols could play a key role in growth of several beneficial bacteria including *Lactobacillus* and *Bifidobacterium spp*. by modulating the growth of other pathogenic bacteria (41, 42). Green tea may change the human intestinal and oral microbiota of healthy individuals (43). Two weeks of green tea liquid usage may increase the *Firmicutes-*to-*Bacteroidetes* ratio, elevate short-chain fatty acids producing genera, and reduce bacterial lipopolysaccharide (LPS) synthesis, effects maintained even after 1 week of washout period (43). In addition to this, green tea is also able to change the salivary and oral epithelium microbiota in humans (43, 44). Mouse studies revealed that green tea extract or its components, EGCG caffeine, and theanine, given for 7 days are also able to modulate the gut, cecum, as well as skin microbiome and metabolites following a single ultraviolet (UV) light stress (41, 45). The strongest effect was observed on *Firmicutes-*to*-Bacteroidetes* ratio after green tea extract, which was decreased after UV light (UV stress vs. green tea extract) (41). A human study also showed that 7 days consumption of green tea extract can lead to a change in metabolite production (46). This study highlights the important role of gut bacteria in the metabolism of green tea extract. In plasma, after 2 h of consumption, green tea extract was metabolized into different components ECGG, GC, and GCG and 16 out of 163 endogenous metabolites were affected including hippurate, taurine serotonin, and 3,4-dihydroxyphenylethylene-glycol (46). This study did not explore the change in the gut microbiota but highlights the potential role of commensals in breaking down green tea extracts. Furthermore, an *in vitro* study also investigated the metabolic fate of EGCG and its influence on gut microbiota and found that EGCG itself can be degraded into several metabolites (47). Microbiome profiling suggested that EGCG treatment increased the growth of several beneficial bacteria such as *Bacteroides* and *Bifidobacterium* and inhibited the growth of pathogenic bacteria *Fusobacterium* and *Enterobacteriaceae* (47). On a metabolic level, 4-phenylbutyric acid was positively or negatively correlated with 11 bacterial genera (*Lachnoclostridium* and *Fusobacterium* are positively related whereas others including *Alistipes* and *Bacteroides* are negatively correlated) (47). 4-Hydroxybenzoic acid had a negative correlation with *Haemophilus* bacterial genera while phenylacetic acid showed positive or negative correlation with bacterial genera (positively with *Fusobacterium* and negatively with *Haemophilus* and *Streptococcus)* (47). Nonetheless, animal and human reports suggest that the degradation of EGCG in the gastrointestinal tract and the function of metabolites should be considered for better understanding the mechanisms of EGCG and immune responses (Figure 1).

## Effect of EGCG On Calcium Signaling in CD4^+^ T Cells

The active component of green tea is EGCG, which is able to ameliorate symptoms and diminish the pathological conditions linked with autoimmune inflammatory diseases in a number of different animal models (1, 8, 20, 35–37, 48, 49). Key cells involved in autoimmune disease promotion or regulation are CD4^+^ T cells and their helper subsets (50). CD4^+^ T helper (Th) cells perform a crucial role in adaptive immune responses (51). These Th cells employ and activate other adaptive immune cells including B cells, and CD8 T cells, as well as other cells involved in the innate immune response (52). Naïve T cells can differentiate into various effector Th cells such as Th1, Th2, Th9, Th17, Th22, T follicular helper (Tfh), and induced regulatory T cells (iTregs) (49, 52–63). These cells secrete different repertoires of cytokines and recruit various arms of the immune response (52, 58). Th1 and Th17 cells are entailed for protection against intracellular pathogens and fungal infections and cancers, whereas Th2 cells are required for protection against helminths (56, 64–66). Th9 and Th22 cells are less well-defined but appear to be important for airway, tumor and skin inflammation, whereas Tfh cells are vital for the activation of B cells and the formation of germinal centers in secondary lymphoid organs (52, 57, 61, 62, 67–77). In contrast, Tregs help to maintain immune homeostasis by suppressing the immune response and preventing reactions against host organs and autoimmunity (51, 52, 78–85).

Recent studies demonstrated that EGCG supplemented in a diet mitigated experimental autoimmune encephalomyelitis (EAE) in a murine model, which was correlated with a lower number of Th1 and Th17 cells and an augmented number of Treg cells in the central nervous system as well as in peripheral lymphoid organs (49, 86). These studies also suggested that EGCG is able to inhibit inflammatory cytokines, namely, IL-12, IL-1β, IL-6, IL-23, and TNF-α. Furthermore, these cytokines were already proven to promote the development of Th1 (IL-12 helps in development and differentiation), Th17 (IL-1β, IL-6, IL-23—all three key cytokines promote the pathogenicity of these cells), and Th9 (TNF-α required for improved differentiation) cells, albeit IL-10 and IL-4 (Th2 cytokines) cytokines were not affected by EGCG (49, 86). Therefore, EGCG is able to modulate the CD4^+^ T cell differentiation (49). Nevertheless, further experimental support for this notion and an in-depth explanation of underlying mechanisms are desirable as Th9 cells are known to induce EAE (54) and EGCG can ameliorate EAE as described above; therefore, examining the impact of EGCG on Th9 cells in detail is required. Nonetheless, EGCG is effective against metabolic syndrome, obesity, and autoimmune arthritis by managing the fine balance of CD4^+^ T cells (37). The multifaced role of green tea and its different components in controlling diverse functions are summarized in Table 1.

**Table 1:** Effects of green tea polyphenol such as EGCG and its different components on immune T cells.

	**Green tea and T cells**	**Effect**	**References**
1	EGCG and CLL B and T cells	Apoptosis	(87)
2	EGCG *in vivo* in MPTP induced Parkinson's disease model	Ratio of CD3^+^CD4^+^/CD3^+^CD8^+^ increased and reduced serum IL-6 and TNF-α	(18)
3	EGCG and mouse T cells as well as Jurkat lymphoblasts	Reduced SOCE expression in T cells	(48)
4	EGCG and arsenic induced inflammation and apoptosis	Decreased the CD4^+^ T cell frequency	(8)
5	EGCG and T cells in aging in Swiss albino mice	Increase frequency of CD3^+^CD8^+^ and reduced IgA, IgE, and IgG1/igG2a and IL-6 and TNF-α	(3)
6	EGCG and Graft-versus-Host Disease	Increased regulatory CD4^+^ T cells and reduced oxidative stress	(17)
7	Green tea metabolites (EGC-M) and T cells activity	Reduced T cell activity by EGCG and EGC Enhanced T cell activity by EGCG-metabolites	(88)
8	EGCG in autoimmune arthritis	Change in the balance in between Th17/Tregs and inhibition of osteoclastogenesis by STAT3	(89)
9	EGCG in autoimmune arthritis	Increase in Tregs	(90)
10	EGCG and vitiligo in human subjects	Reducing the inflammatory cytokines from T cells by JAK2 pathway	(91)
11	Green tea EGCG and human mast leukemic cell line (HMC-1)	Modulation of the NF-κB/ERK1/2/MAPK signaling pathway	(92)
12	EGCG and autoimmune arthritis	Restraint STAT3 and HIF-1α with Th17/Treg ratio	(1, 21)
13	EGCG and obesity and autoinflammatory arthritis	Change in balance in CD4^+^ T cells subsets	(37)
14	Green tea and chronic lymphocytic leukemia	Change in Tregs and reduced IL-10 and TGF-β in serum	(93)
15	EGCG and T cells differentiation in EAE model	Reduced IL-6 and IL-6R and increase soluble gb130 in plasma from EAE mice	(49, 86)
16	EGCG and increased inflammation	High dose of EGCG leads to increased IL-6, IL-1β, PGE2, and deceased IL-4	(36)
17	EGCG and proliferation of T cells	Impaired IL-2/IL-2R signaling and IL-2 signaling, cell cycle and proliferation	(5, 94)
18	EGCG and T cell receptor signaling	Inhibition of ZAP-70 kinase signaling	(95)
19	EGCG and TCR binding for HIV	TCR CD4 binding with HIV-1 protein	(96)
20	Green tea EGCG and neuroprotection	NFκB inhibition in EAE model	(97)
21	Green tea and T cell apoptosis	Increased apoptosis in peripheral T lymphocytes in adult leukemia patients	(98)

In several diseases, EGCG affects the outcome by modulating the function of T cells. Differential effects of EGCG are observed on the proliferation of B and T cells from B-cell chronic lymphocytic leukemia (CLL) patients compared with healthy controls in a dose-dependent fashion (87). T or B cells are more prone to apoptosis in CLL patients compared with healthy controls (87). EGCG is shown to inhibit murine CD4^+^ T cell proliferation and induces apoptosis *in vitro* (Table 1) (5, 48, 94). However, EGCG in the gut of human and mice can also be converted into different metabolites, which could exert different effects on immune T cell functions. Kim et al. reported that 11 EGCG metabolites have a differential effect on murine CD4^+^ T cells compared with EGCG (88). EGCG and EGC green tea catechins decrease ATP levels, thus suggesting an inhibitory role in T cell activation. However, EGC metabolites (7 out of 11 metabolites) increased ATP levels compared with control and EGCG, thus reflecting activating effects on T cell functions (88). These results highlight the importance of gut bacteria on differential outcome of EGCG and their metabolites for regulating the functions of immune T cells. This could be a potential explanation why different people observe such heterogenic effects. Clearly, caution is warranted during interpretation of findings.

After engagement of the T cell receptor (TCR) with its cognate antigens leads to an activation of T cells, further activation triggers an increase in intracellular Ca^2+^ levels that is needed for the essential physiological functions of T cells such as gene expression, proliferation, cell motility, and cytokine production (99, 100). In naïve or resting T cells, Ca^2+^ accumulates in the endoplasmic reticulum (ER) of the cells and levels of Ca^2+^ are gauged by stromal cell-interaction proteins (STIM) 1 and 2 (101). Once TCRs are activated (after antigenic stimulation), inositol trisphosphate (IP3) is produced followed by binding to IP3 receptors expressed on the ER and results in the release of intravesicular Ca^2+^ into the cytosol (102, 103). The calcium store exhaustion stimulates Ca^2+^ influx across the plasma membrane of the T cells, a process called store-operated Ca^2+^ entry (SOCE) (104–106). SOCE results from assembly of calcium release-activated calcium (CRAC) channel protein 1, which is encoded by the *Orai1* gene with the ER Ca^2+^ sensing proteins STIM1 and STIM2 (106). Orai1-mediated Ca^2+^ influx in T cells depends on a negative membrane potential delivering the electrical driving force for Ca^2+^ entry into the cells (100, 106). The membrane is polarized by opening of K^+^ channels and depolarized by opening of Na^+^ channels. Two K^+^ channels are known to be activated upon Ca^2+^ influx—the voltage-gated K^+^ channel (K_V_1.3) and the calcium-activated K^+^ channels (K_Ca_3.1) (107–111). Negative feedback provided to these K^+^ channels is established by the transient receptor potential cation channel, subfamily M, member 4 (TRPM4), which mediates Na^+^ entry, thus depolarizing the membrane and curtailing Ca^2+^ entry through Orai1 (112). Further, the cell membrane potential also affects Cl^−^ flux through Cl^−^ channels and thus cell volume. When cells are exposed to hypotonic conditions, this results in swelling of T cells and Cl^−^ channels start to operate. Cell swelling triggers the efflux of Cl^−^ and eventually water from the cells, which returns the cell to its normal volume (102). The movement of Ca^2+^, K^+^, Na^+^, and Cl^−^ ions ultimately affects the release of Ca^2+^; thus, regulating the performance of these ion channels would help to shape the signaling in T cells pivotal in development of Th cells and function (102).

The significance of ion channel function in T cells is mostly derived from genetic studies performed in murine models using either ion channel-specific gene knockout or siRNA knockdown (103). STIM1/2 or Orai1 (CRAC) knockout murine models have improved our knowledge on how these proteins participate in defective T cells' development contemplating the functions of these proteins in Ca^2+^ signaling (100, 102, 113). Furthermore, patients with mutations in these genes also have profound defects in T cell development and function and are therefore immunodeficient (104). In mice, depletion of these genes disrupts the production of IL-2, IFN-γ, IL-17 and TNF-α, and thereby inhibits development of all Th cell classes (106, 114). The knockout of K_Ca_3.1 or K_V_1.3 results in the reduction of Ca^2+^ influx upon stimulation of T cells (108, 109, 111). Inflammatory cytokines, namely, IFN-γ and IL-17, are attenuated, indicating a defect in the development and/or function of these inflammatory Th cell types (115). However, Treg development and function appear normal and these mice are resistant to autoimmune disorders (108). Deletion of K_Ca_3.1 protects mice from developing colitis whereas K_V_1.3 gene deletion prefers T cells toward immunoregulatory in function and renders the gene knockout mice impervious to autoimmune encephalomyelitis (109, 116, 117). Therefore, K^+^ channels are differentially required for the development and function of the various Th cell types. In addition, the K_V_1.3 channel is specifically upregulated in Th17 cells and is required for its activation and cytokine production (108). With regard to Na^+^ channels, gene array analysis indicates that TRPM4 is expressed more in Th2 compared to Th1 cells (112). Experiments performed in T cells for TRPM4 gene silencing using siRNA increases Ca^2+^ influx in Th2 cells, whereas it decreases Ca^2+^ influx in Th1 cells (102, 112). It also affects the T cell cytokine production of IL-2, IL-4 and IFN-γ in addition to cell mobility. However, the mechanisms underlying those effects are incompletely understood because the expression of Th1 and Th2 transcription factors Tbet and GATA3 are not affected, respectively (112, 118). In summary, these studies suggest that ion channels are differentially involved for the development and function of Th cell subtypes.

So far, only few studies were performed to understand the influence of green tea on SOCE pathway in CD4^+^ immune T cells (5, 48, 92, 94, 119–121). Other immune cells such as mast cells were given the treatment of EGCG in varying doses, which could inhibit the functions of mast cells such as degranulation, leukotriene C4 secretion, and SOCE (Ca^2+^ flow) through mitochondrial calcium dysfunctions (119). In human Jurkat T cells, it is demonstrated that EGCG is capable to diminish the calcium influx (48, 120). Recently, one study in murine primary CD4^+^ T cells suggested that EGCG is able to inhibit the SOCE in a dose-dependent fashion and affects cell proliferation and apoptosis (48). Thus, EGCG inhibits Ca^2+^ influx in immune cells including T cells.

## EGCG Controls miRNAS Expression in Cancer and Immune T Cells

MicroRNAs (miRNAs) are non-coding very small (single-stranded ~19–23 nucleotides) RNA molecules that regulate at least one third of genome (gene expression) at the post-transcriptional level (122). These miRNAs are instructed by host genes and appear to present in both intronic and exonic regions of protein-coding genes as well as in non-coding genes (123–125). In general, the process of miRNAs biogenesis begins in the nucleus of a T cell or other cell types from a primary miRNA (pri-miR) transcript, which changes into a secondary structure comprising either one or more hairpin loops or lollipop structures (126–128). These hairpin loops or lollipop structures are identified and processed by the microprocessor complex enzymes constituted of DiGeorge syndrome critical region 8 (DGR8) and Drosha (127, 129, 130). This enzymatic process yields a stem loop precursor miRNA (pre-miR) that consists of roughly 60–70 nucleotides. The pre-miR is transported to the cytoplasm by another protein called exportin-5 where it undertakes a secondary processing stage by another RNase III enzyme called Dicer yielding a RNA duplex of 19–23 nucleotides (130). This double-stranded RNA duplex is amalgamated into the RNA-induced silencing complex (RISC), where one of the RNA strands results in degradation while the subsequent RNA strand forms the mature miRNA involved in a post-translational process (131). Overall, most of the mature miRNA attaches to the 3′ UTR untranslated region (UTR) of its target mRNA transcript. However, in some instances, mature miRNAs could also attach to the 5′ UTR and the protein coding region of the gene (128). Once the binding is completed, then RISC either inhibits the translational process or degrades the targeted mRNA, thus decreasing protein expression (123, 132). Dysregulated miRNAs are involved in several pathological conditions including autoimmunity, infection, and cancer (125).

Various studies suggested that EGCG is able to upregulate several different miRNAs and also downregulates several of them; however, most of the studies focused on the miRNAs that were upregulated after green tea and its components such as EGCG (Table 2), thus affecting gene regulation and the respective cell functions such as cell proliferation, apoptosis, etc.

**Table 2:** Effects of green tea and its different componment on miRNAs expression in cancer and T cells.

**SN**	**Cells**	**Dysregulated miRNAs**	**Functions**	**References**
1	Non-small-cell lung cancer (NSCLC) treated with EGCG	hsa-miR-485-5p	Inhibits cell growth and cell apoptosis RXRα gene	(15)
2	EGCG and EGC in hypertensive model	miR-126a-3p and miR-150-5p	Hypertension SP1/AT1R pathway	(133)
3	EGCG on mouse CD4^+^ T cells and human Jurkat lymphoblasts	miR-15b-5p	Calcium functions–SOCE pathway	(48)
4	EGCG IL-1β-stimulated human osteoarthritis chondrocytes	miR-140-3p and miR-199a-39	Anti-arthritic of EGCG by ADAMTS5 and downregulation of COX2 pathway	(16, 19)
5	EGCG and hepatic stellate cells (HepG2)	miR-221, miR-181a, and miR-10b	OPN mRNA degradation and protective in liver injury	(134)
6	EGCG and Mouse lung adenocarcinoma	miR-449c-5p	Myb pathway regulation	(22)
7	EGCG and melanoma cells	miR-let-7b	Laminin receptor signaling	(135)
8	EGCG and osteosarcoma	miR-1	Cell growth	(34)
9	EGCG and tobacco carcinogen-induced lung tumor in A7J mice	miR-210 and other miRNAs	HIF-1α, cell growth AKT, NF-κB, MAP kinase, and cell cycle	(11)
10	EGCG and SH-SY5Y and SK-N-DZ	miR-7-1	Induction of apoptosis	(10)
11	Human lung cancer and EGCG or green tea	miR-7	Apoptosis induction and inhibition of proliferation	(136)
12	Polyphenon-60 and MCF-7	miR-21 and miR-27	Downregulation of the tumor suppressor gene-tropomyosin-1	(137)
13	EGCG and human hepatocellular carcinoma HepG2	miR-16	Induction of apoptosis (by downregulating the apoptotic protein BCL2)	(9)

**Figure 2:**
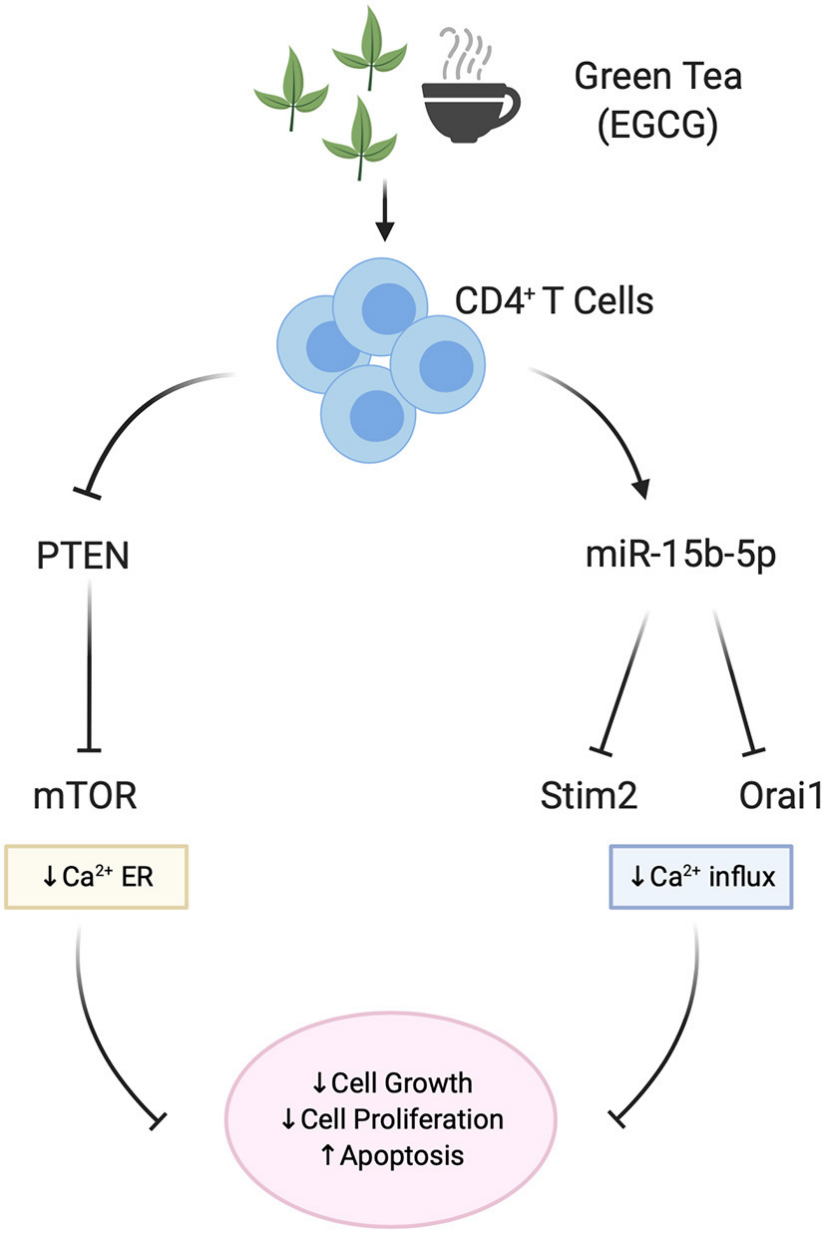
Effect of EGCG on miRNA and Ca^2+^ signaling in T cells. EGCG is able to upregulate miR-15b-5p, thus decreasing Calcium influx (SOCE) proteins Orai1/STIM2. As a result, Ca^2+^-sensitive functions of T cells such as cell proliferation and cell growth in mice CD4^+^ T cells are blunted. As shown in human Jurkat lymphoblasts, EGCG also downregulates the PTEN/mTOR pathway and mitochondrial potential in addition to the Calcium influx, thus affecting the cell growth and proliferation.

The contribution of miRNAs in the modification of Th cell development and function by EGCG has recently been uncovered (48, 138). One study suggested that EGCG upregulates miR-15b with subsequent suppression of Orai1/STIM2 protein synthesis and blunted SOCE (48). This study suggested that miR-15b could be a powerful post-transcriptional regulator of calcium entry and thus of calcium-sensitive functions of T cells (Figure 2).

EGCG differentially augments the expression of several miRNAs (Table 2) that are involved in the NF-κB inflammatory pathway (11), the retinoid X receptor α (RXRα) signaling pathway (15), downregulation of apoptotic protein (10) such as BCL2 (9), downregulation of tumor suppressor genes tropomyosin-1 (137), laminin receptor signaling (135), Myb pathway modulation (22), Cox2 signaling (16, 19), and calcium signaling (139). As scientific advances are developed in miRNA and tea research, an increasing number of molecular effects are recognized due to miRNA regulation. miRNAs induced by green tea have wide-ranging beneficial effects: tumor suppression by negatively regulating gene expression of oncogenic factors, reduction in hypertension and neurodegeneration, and improvement in arthritis (10, 16, 19, 34, 133, 137). Generally, green tea is safe to consume even at high concentrations. Thus, if the cytotoxic effects of green tea can be associated to a specific miRNA, it is plausible that treatments targeting the overexpressed miRNA could be harnessed for treatment of several pathologies. Prospective studies are needed to define which miRNAs could be exploited for therapeutic applications.

## Concluding Remarks and Summary

In recent decades, there is a growing trend in the use of alternative therapies, and plant-based medicinal phytochemicals are among the most suited in inflammatory diseases. Therefore, an appropriate record of traditional herbal medicine in combination with modern scientific/pharmacological investigation is needed to corroborate or disprove the medicinal properties of these countless traditional Phytotherapies used in ancient times in many countries throughout the world (140). In this regard, EGCG from green tea is one of the substances with several historical beneficial effects on various disorders such as cancer, metabolic diseases, and inflammation (89). In CD4^+^ T cells, it appears that EGCG is a powerful regulator of Ca^2+^ signaling by miRNA expression and, thus, by modification of gene expression at the post-transcriptional level. Therefore, it is worth exploring the potential mechanisms of polyphenols in the regulation of other biological processes in addition to immune response.

## Author Contributions

YS, MS, and FL have collected the literature and wrote the review. All authors contributed to the article and approved the submitted version.

## Conflict of Interest

The authors declare that the research was conducted in the absence of any commercial or financial relationships that could be construed as a potential conflict of interest.
